# Viperin inhibits interferon-γ production to promote *Mycobacterium tuberculosis* survival by disrupting TBK1-IKKε-IRF3-axis and JAK-STAT signaling

**DOI:** 10.1007/s00011-024-01873-w

**Published:** 2024-04-16

**Authors:** Yao Liang, Yun Liang, Qi Wang, Qianna Li, Yingqi Huang, Rong Li, Xiaoxin Pan, Linmiao Lie, Hui Xu, Zhenyu Han, Honglin Liu, Qian Wen, Chaoying Zhou, Li Ma, Xinying Zhou

**Affiliations:** 1 Institute of Molecular Immunology, School of Laboratory Medicine and Biotechnology, Southern Medical University, Guangzhou, 510515 China; 2 Key Laboratory of Infectious Diseases Research in South China (Southern Medical University), Ministry of Education, Guangzhou, China

**Keywords:** Viperin, IFN-γ, TBK1-IKKε-IRF3-axis, JAK-STAT signaling, *Mycobacterium tuberculosis* (Mtb)

## Abstract

**Objectives and design:**

As an interferon-inducible protein, Viperin has broad-spectrum antiviral effects and regulation of host immune responses. We aim to investigate how Viperin regulates interferon-γ (IFN-γ) production in macrophages to control *Mycobacterium tuberculosis* (Mtb) infection.

**Methods:**

We use Viperin deficient bone-marrow-derived macrophage (BMDM) to investigate the effects and machines of Viperin on Mtb infection.

**Results:**

Viperin inhibited IFN-γ production in macrophages and in the lung of mice to promote Mtb survival. Further insight into the mechanisms of Viperin-mediated regulation of IFN-γ production revealed the role of TANK-binding kinase 1 (TBK1), the TAK1-dependent inhibition of NF-kappa B kinase-epsilon (IKKε), and interferon regulatory factor 3 (IRF3). Inhibition of the TBK1-IKKε-IRF3 axis restored IFN-γ production reduced by Viperin knockout in BMDM and suppressed intracellular Mtb survival. Moreover, Viperin deficiency activated the Janus kinase (JAK)-signal transducer and activator of transcription (STAT) signaling pathway, which promoted IFN-γ production and inhibited Mtb infection in BMDM. Additionally, a combination of the anti-TB drug INH treatment in the absence of Viperin resulted in further IFN-γ production and anti-TB effect.

**Conclusions:**

This study highlights the involvement of TBK1-IKKε-IRF3 axis and JAK-STAT signaling pathways in Viperin-suppressed IFN-γ production in Mtb infected macrophages, and identifies a novel mechanism of Viperin on negatively regulating host immune response to Mtb infection.

**Supplementary Information:**

The online version contains supplementary material available at 10.1007/s00011-024-01873-w.

## Introduction

Tuberculosis (TB) caused by *Mycobacterium tuberculosis* (Mtb) infection is a worldwide disease that affects one-third of the world's population [1]. Mtb is an intracellular pathogen living in macrophage, which is the first line of host defense against the infection [2]. Multiple host molecules and their associated signaling pathways in macrophages are involved in the regulation of Mtb infection [3]. The in-depth study of the regulation of host factors in macrophages on the immune response to Mtb infection has become an urgent need for the development of host-directed therapies for TB.

Interferons (IFNs) are a family of pleiotropic cytokines that play important roles in the host defense against Mtb infection. Type I IFNs (IFN-α and IFN-β) can control Mtb infection as well as cause damage to host cells. Accumulating studies have shown that IFN-γ, the sole type II IFN, is pivotal in anti-Mtb immune response of both mice and humans [4, 5]. Although IFN-γ production from NK and NKT cells as well as CD4^+^ and CD8^+^ lymphocytes play a primary function in anti-Mtb immune response, macrophages are capable of producing IFN-γ and promoting host defense mechanisms that control Mtb infection [6]. IFN-γ signaling leads to autophagy, phagosome maturation, programmed death activation of host cells, ROS generation and other mechanisms to act against Mtb [7].

Accumulating studies have investigated the signals that are required for IFN-γ production in macrophages. Interferon regulatory factor 3 (IRF3) signaling is an important pathway that not only regulates type I IFNs, but also IFN-γ production. IRF3 phosphorylation is associated with multiple upstream kinases including the TANK-binding kinase 1 (TBK1) and the TAK1-dependent activation of inhibitor of NF-kappa B kinase-epsilon (IKKε) [8, 9]. Whether TBK1-IKKε-IRF3 signaling plays a role in regulating IFN-γ production in Mtb- infected macrophages remains unknown. The binding of IFN-γ to its receptor IFN-γR1 and IL-10R2 can activate a subset of downstream signaling pathways, particularly the Janus kinase (JAK)-signal transducer and activator of transcription (STAT), resulting in gene transcription within the target cells to exert host defense function [10]. The JAK-STAT signaling pathway could also drive the regulation of the next wave of transcription of ISGs and lead to the production of IFN-γ [11]. How these signaling pathways cooperate with each other to regulate IFN-γ secretion in Mtb infected macrophages deserves further study.

Viperin (also known as Rsad2, Cig5, or Vig1) is an IFN-stimulated gene (ISG) encoded protein. It could interact with a large number of viral and host proteins to elicit a wide spectrum of antiviral activities [12]. Recently, Viperin has been found to increase lipogenesis and glycolysis to promote metabolic reprogramming and facilitate cancer progression [13]. Through regulating innate immune response, Viperin can inhibit macrophage polarization and secretion of M1 and M2 cytokines [14]. Viperin also participates in different cell signaling of the immune system by promoting NF-κB and AP-1 signaling in T cells [15]. Chikungunya virus (CHIKV)-infected Viperin deficient mice showed an increased Th1 profile of CD4^+^ T cells, enhanced IFN-γ stimulation by APCs, and increased numbers of inflammatory monocytes [16]. Viperin promoted TLR7- and TLR9-mediated production of type I IFN by pDCs to exert its antiviral function [17]. Previously, we found some of ISGs are vital in regulating immune response to Mtb infection, and especially Viperin could negatively regulate the activation of macrophages and dendritic cells against Mtb infection [18, 19]. However, the role of Viperin in IFN-γ production and Mtb infection in macrophages remains to be studied.

Our findings reveal the alternative function of Viperin on IFN-γ production in regulation of Mtb infection in macrophages, reinforce the idea of blocking IFN-γ production by Viperin deficiency as a host-directed therapy against TB, and raise the possibility that blocking a specific downstream step in IFN-γ signaling could protect macrophages from Mtb infection.

## Materials and methods

### Ethics statement

This study was performed according to the recommendations of the International Committee of Medical Journal Editors and approved by the Ethics Committee of the Southern Medical University. All animal experiments in this study were conducted in accordance with the National Institutes of Health Guidelines for the Care and Use of Laboratory Animals. All protocols were approved by the Medical Ethics Committee and Biosafety Management Committee of Southern Medical University. A Highly pathogenic microorganism laboratory management commitment letter was approved by Southern Medical University.

### Mice

C57BL/6J mice were purchased from the Laboratory Animal Center of Southern Medical University (Guangzhou, China). Viperin deficient (*Rsad2*^−/+^) mice on a C57BL/6J background were derived by Cyagen Biosciences Inc. (serial number: KOCMP-02845-*Rsad2*, Nanjing, China). All mice were maintained in the Laboratory Animal Center of the Southern Medical University under specific pathogen–free conditions.

### Differentiation of mouse macrophages in vitro

Bone marrow-derived macrophages were obtained from 4 to 6 week-old *Rsad2*^+*/*+^ and *Rsad2*^−/−^ female mice. The cells were cultured using DMEM complete medium containing 100 ng/ml mGM-CSF (Pepro Tech, USA) and 10% FBS (Corning, NY, USA) in 5% CO_2_ incubator at 37 ℃. On day three and five, half of the above complete medium is refreshed. After seven days of culture, we digested the cells with pancreatic enzyme and cultured them in 5% CO_2_ cell culture incubator at 37 ℃ for another 24 h before they could be performed.

## Mtb culture and infection

Mtb H37Rv (American Type Culture Collection, NO.7294) were cultured in 7H9 broth medium (Becton Dickinson, New Jersey, USA) containing 10% OADC (0.06% (v/v) oleic acid (SIGMA, St. Louis, MO, USA), 5% albumin (SIGMA, St. Louis, MO, USA), 100 mM glucose (GHTECH, Guangzhou, China), 0.003% catalase (SIGMA, St. Louis, MO, USA), and 145 mM NaCl (GHTECH, Guangzhou, China) in 5% CO_2_ incubator at 37 ℃. Mtb should be passaged every month to maintain the viability and virulence. A single bacterial suspension was prepared for infectious experiments. The concentration of a single bacterial suspension was calculated by detecting the absorbance at 600 nm wavelength (OD_600_) by universal microplate reader (Thermo Fisher Scientific, Carlsbad, CA, USA). BMDMs were infected according to the multiplicity of infection (MOI) required for the experiment.

### Treatment of reagents in macrophages

BMDMs were pretreated with neutralizing antibody of IFN-γ (7.5 µg/mL) (Selleck, Houston, USA), inhibitor of BX-795 for TBK1/IKK-ε (2 µM) (Merck KGaA, Germany), inhibitor of Bay-985 for IRF3 (200 nM) (Merck KGaA, Germany), inhibitor of Abrocitinib for JAK1 (200 nM) (Merck KGaA, Germany), inhibitor of Fludarabine for STAT1 (30 µM) (Merck KGaA, Germany) and dimethylsulfoxide (DMSO) as solvent control for corresponding processing time before H37Rv infection. Pre-processing time and other details of the reagents are shown in Supplementary Table 1.

### RNA extraction and quantitative real-time PCR

Total cellular RNA was extracted from BMDMs by TRIzol-based method, and RNA concentration was measured by NanoDrop 2000 (Thermo Fisher Scientific, Carlsbad, CA, USA). Then cDNA synthesis and reverse transcription experiments were carried out using cDNA synthesis kit HonorTM II 1st Strand cDNA Synthesis SuperMix (Novogene, Beijing, China). The abundance of mRNA of individual genes was quantified by real-time PCR using a SYBR® Premix Ex TaqTM II (Tli RNaseH Plus) (TaKaRa, Beijing, China) on a LightCycler 480 thermocycler (Roche, Basel, Switzerland). The reaction procedure of qPCR was adjusted according to the three-step method, which was predenaturation at 95 ℃ for 2 min, 40 cycles at 95 ℃ for 15 s, 40 cycles at 60 ℃ for 15 s, 40 cycles at 68 ℃ for 20 s, and finally stored at 4 ℃. All PCR products were normalized to GAPDH or β-actin transcript, then relative gene expression abundance was calculated using the 2^−ΔΔCT^ method and expressed as fold change. The complete primer sequences are shown in Supplementary Table 2.

### Enzyme-linked immunosorbent assay (ELISA)

The cultured cells were collected into 1.5 mL EP tube, and the residual H37Rv was filtered by 0.22 μm microporous filter. The extracellular secretion of IFN-α、IFN-β and IFN-γ was detected by corresponding ELISA kit (IFN-α: Thermo Fisher Scientific, USA; IFN-β and IFN-γ: MultiSciences, Hangzhou, China). All experimental steps are strictly in accordance with the reagent instructions provided by the corresponding manufacturer. The wavelength of 450 nm and 630 nm was set with the Microplate Reader (TECAN SPARK, Austria) to read the corresponding OD value, and the corresponding concentrations of IFN-α, IFN-β and IFN-γ were calculated according to the standard curve.

### Immunohistochemical fluorescence analysis

4–6 week-old *Rsad2*^*−/−*^ and *Rsad2*^+*/*+^ C57BL/6J female mice with similar weight were exposed to 1 × 10^7^ colony-forming units (CFUs) of H37Rv in an Inhalation Exposure System (Glas-Col, USA), which delivers about 200 bacteria per lung to the animal. Four weeks after infection, the lungs and spleens were dissected out and immersed in formaldehyde. The corresponding lung and spleen tissues were prepared into paraffin sections (xylene 1 for 15 min (Sinopharm, Beijing, China), xylene 2 for 15 min, anhydrous ethanol 1 for 5 min (Sinopharm, Beijing, China), anhydrous ethanol 2 for 5 min, 95% ethanol for 5 min, 85% ethanol for 5 min, 75% ethanol for 5 min, and finally rinsed with water). With the method of ice cut fixed after drying. 1 × citric acid (pH 6.0) (Sinopharm, Beijing, China) was used as the repair solution for antigen repair. Endogenously blocked by incubating 3% H_2_O_2_ (Sinopharm, Beijing, China) with water for 20 min at room temperature. The serum from the same source as the second antibody was incubated at 37 ℃ for 30 min for sealing. The relevant primary antibody was diluted with goat serum (Thermo Fisher Scientific, Carlsbad, CA, USA) and incubated at 4 ℃ overnight. Secondary antibody was incubated (HRP labeled secondary antibody was used) (Pinuofei, Wuhan, China). PBS-T (Pinuofei, Wuhan, China) was used to prepare secondary antibody and incubated at 37 ℃ for 1h. TSA reagent (Pinuofei, Wuhan, China) was incubated at 37 ℃ for 30 min and washed three times with PBS. Repeat the antigen repair step to the TSA incubation step. DAPI working droplets (Pinuofei, Wuhan, China) were added to the tissue, incubated at room temperature for 5 min, and then washed the tissue slice with PBS (Pinuofei, Wuhan, China) for 3 times for 5 min each time. After drying, anti-fluorescent tablet was added to the tissue and the cover glass was covered. The fluorescence slides were stored at 4 ℃ away from light or observed in pathological biopsy scanner (3DHISTECH Pannoramic SCAN II, Hungary). The obtained images were scanned and analyzed using Image J Software, and the results were analyzed using Pearson correlation coefficient. The details of the antibodies are shown in Supplementary Table 3.

### Colony-forming unit (CFU) assays

BMDMs were seeded in 12-well plates according to 5 × 10^5^ per well and were infected with H37Rv of MOI = 5 for 1 h. The cells were then with PBS for 3 times at a volume of 1 mL per well to remove excess extracellular bacteria, and complete medium was added to culture at different time points. BMDMs were lysed in ddH_2_O containing 0.01% Triton X-100 (Solarbio, Beijing, China). After 0.01% TritonX-100 was used to dilute the lysed cells (100-fold dilution at 0 h, 1000-fold dilution at 24 and 48 h). 50 μl of the dilution was thoroughly mixed and inoculated on 7H10 agar plate (Becton Dickinson, New Jersey, USA). Use a coating stick to spread the bacterial solution evenly and repeat 8 to 10 times per set. After coating, seal it and place the 7H10 agar plate upside down in 5% CO_2_ incubator at 37 ℃ for culture. After 3–4 weeks, the number of colonies can be counted. In order to detect the direct killing effect of drugs on bacteria, we conducted CFU assay. According to the drug dosage of the experimental protocol, 25μl drug diluent was thoroughly mixed with 25 μl bacterial solution (containing 5000 bacteria) and incubated for a specified time, then inoculated on a 7H10 AGAR plate (Becton Dickinson, New Jersey, USA). Use a coating stick to spread the bacterial solution evenly and repeat 8–10 times per set. After coating, seal it and place the 7H10 agar plate upside down in 5% CO_2_ incubator at 37 ℃ for culture. After 3–4 weeks, the number of colonies can be counted.

### Western Blot analysis

After the medium was discarded, 1 × PBS was added to wash the cells for three times. The protein lysate [composed of 455 mM Tris HCl (pH 6.8) (Sangon Biotech, Shanghai, China), 41.6 mM SDS (Zhuosheng Biotech, Shanghai, China), 26.9 μM bromophenol blue (Solarbio, Beijing, China), 30% (v/v) glycerol (SIGMA, St. Louis, MO, USA), and 10 μM dl-dithiothreitol (DTT) (SIGMA, St. Louis, MO, USA)] was added according to the volume of 200 μL per well of the 12-well plate. The sample was then placed in a metal dry heater at 95 ℃ and heated for 10 min to fully denature the protein. According to the experimental requirements, different concentrations of separation glue (8% and 10%) and 5% laminated glue were prepared. The protein was transferred to PVDF membranes (Merck KGaA, Germany) after electrophoresis and electrolysis. The target protein was cut out according to the protein marker (BIORAD, Hercules, CA, USA) and the membranes were blocked in 5% (w/v) BSA (SIGMA, St. Louis, MO, USA) in PBS-T for 1 h at room temperature. According to the instructions of the primary antibody of the target protein, the antibody was diluted in the corresponding proportion and then added. The membranes were slowly shaken at 4 ℃ overnight. The membranes was shaken with PBST for 10 min and repeated three times before adding HRP-conjugated goat anti-rabbit or goat anti-mouse secondary antibody (Cell Signaling Technology, Danvers, MA, USA) for 1 h at room temperature. Then PBS-T was added to wash the second antibody, the washing was repeated three times for 10 min each time. The target protein bands were visualized by enhanced chemiluminescence (ECL, Thermo Fisher Scientific, USA) on FluorChem Systems (ProteinSimple, USA). All strips of immunoblotting were scanned by Image J software (National Institutes of Health) for quantitative density values, and normalized to 1.0 with β-actin as a control. All the antibodies used in this project are listed in Supplemental Table 3.

### Confocal microscopic analysis

BMDMs were inoculated in 24-well plates containing polylysine-treated slide (Thermo Fisher Scientific, Carlsbad, CA, USA) with the number of 1 × 10^5^ per well. After PBS was added to clean the residual medium, 4% paraformaldehyde was added and placed overnight at 4 ℃ away from light. The slides were shaken with PBS for 10 min and repeated three times before 0.2% Triton-X-PBS for 5 min. Then PBS containing 0.05% Tween was added to wash the slide for three times before blocking with PBS-T containing 10% (w/v) BSA (SIGMA, St. Louis, MO, USA) for 30 min at room temperature. According to the instructions of the primary antibody of the target protein, the antibody was diluted in the corresponding proportion and then added. The slides were slowly shaken at 4 ℃ overnight. After washing the slides with 0.05% PBS-T for three times, corresponding fluorescent secondary antibody was incubated for 30 min at room temperature in the dark. Then slides were shaken with 0.05% Tween-PBS for 10 min and repeated three times before PBS for 3 min. DAPI (Sangon Biotech, Shanghai, China) was added to stain the nucleus, and left for 10 min at room temperature. Then the slides were washed for three times with 1 × PBS. The slides were sealed with ProLong Gold Antifade reagent (Invitrogen, MO, USA) and observed under Zeiss Axiovert LSM 880 instrument (Zeiss, Gottingen, Germany). According to the staining range of DAPI, the nuclei were circled and the average fluorescence intensity data was obtained.

### Cell viability assays

Cell viability was measured with TransDetect CCK8 kit (TransGen Biotech, China). BMDMs were inoculated in 96-well plates with the number of 1 × 10^4^ per well. After different treatments at different times, the medium containing CCK-8 solution was replaced and cultured at 37 ℃ for about 1 h. The Varioskan Flash (Thermo Fisher Scientific, Carlsbad, CA, USA) measured the absorbance at 450 nm and 630 nm within 30 min.

### Statistical analysis

Statistical analysis was performed using Prism 7.0/SPSS 22.0 software. At least three independent repeated experiments were conducted for the measurement data results, and the measurement data were expressed as mean ± SD. One-Way ANOVA or Independent-samples *t*-test were used for comparison. *p ≤ 0.05; **p ≤ 0.01; ***p ≤ 0.001 were considered statistically significant.

## Results

### Viperin deficiency promotes IFN-γ production to inhibit Mtb infection in macrophages

**Fig. 1:**
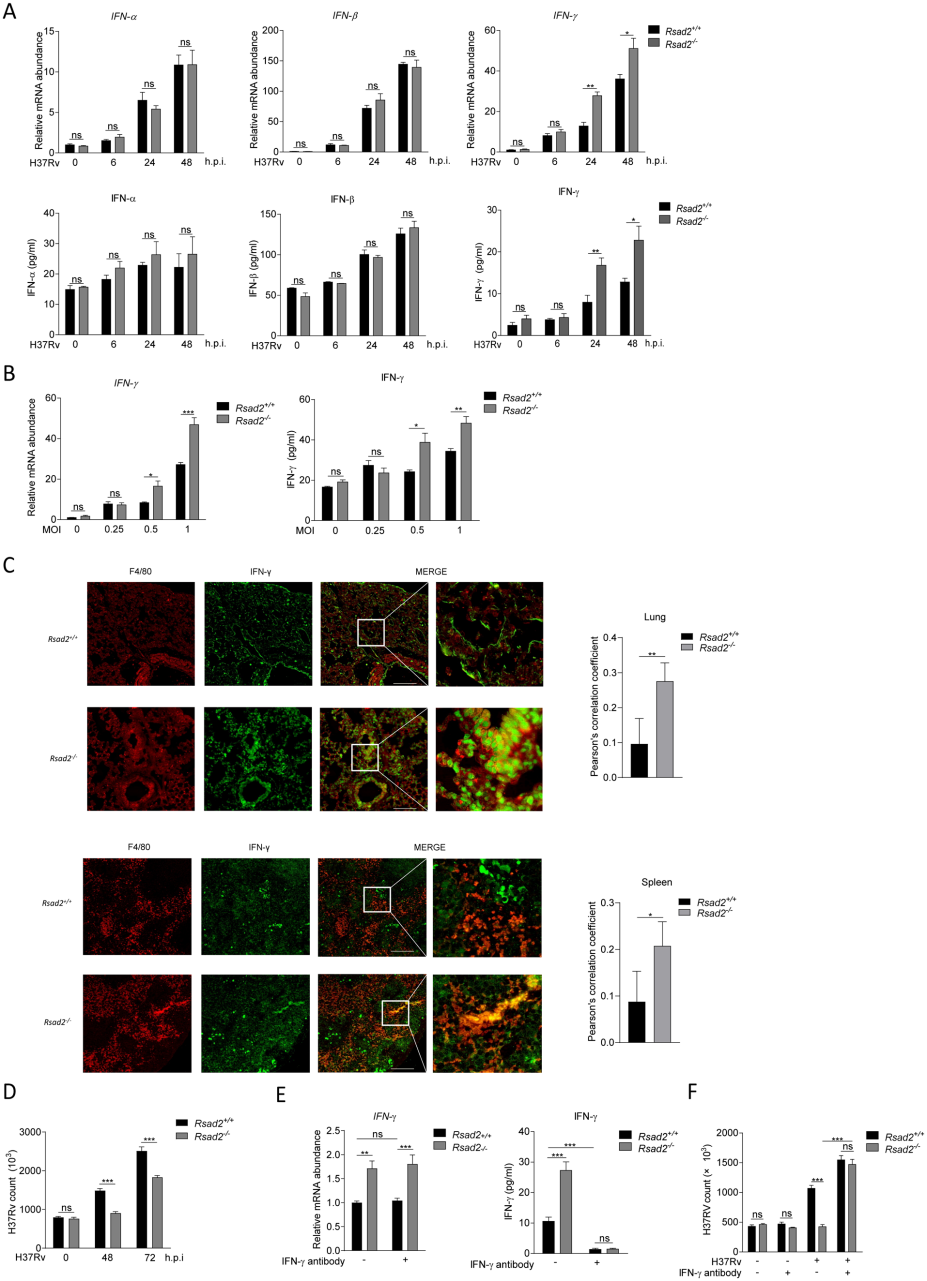
Viperin deficiency promotes IFN-γ production to inhibit Mtb infection in macrophages. **A** Intracellular mRNA and secretion of IFN-α, -β and -γ were detected in *Rsad2*^+/+^ and *Rsad2*^−/−^ BMDMs at 0, 6, 24 and 48 h of Mtb infection at an MOI of 1 by qRT-PCR and ELISA analyses. **B** Intracellular mRNA and secretion of IFN-γ were detected in *Rsad2*^+/+^ and *Rsad2*^−/−^ BMDMs at 48 h of Mtb infection at MOI of 0, 0.25, 0.5 and 1 by qRT-PCR and ELISA assays. **C** *Rsad2*^+/+^ and *Rsad2*^−/−^ mice (five mice per group) aged from 6 to 8 weeks were infected with about 200 colony-forming units (CFUs) of the Mtb strain by aerosol and were analyzed 4 weeks later. Left: F4/80 and IFN-γ protein in the lungs and spleens of *Rsad2*^+/+^ and *Rsad2*^−/−^ mice was assessed by dual immunofluorescence technique. Right: Dual immunofluorescence maps were quantified by the Pilson coefficient. At least five visual fields were randomly from each slide in each group for analysis. Original magnification: × 40. Scale bars, 100 μm. **D** CFU counts were determined to analyze Mtb survival in *Rsad2*^−/−^ BMDMs at 48 and 72 h with Mtb infection at MOI of 5. **E** Intracellular mRNA and secretion of IFN-γ were detected in *Rsad2*^+/+^ and *Rsad2*^−/−^ BMDMs pretreated with 7.5 μg/mL IFN-γ neutralizing antibody for 2 h before 48 h of Mtb infection at MOI of 1 by qRT-PCR and ELISA analyses. **F** CFU counts were determined to analyze Mtb survival at MOI of 5 after 48 h of infection in *Rsad2*^+/+^ and *Rsad2*^−/−^ BMDMs that were pretreated for 2 h with 7.5 μg/mL IFN-γ neutralizing antibody. **A**, **B**, **E** Data were presented as fold change in mRNA abundance relative to that in (**A**, **C**) uninfected controls or (**F**) untreated controls of *Rsad2*^+/+^ BMDMs. β-Actin served as an internal reference. **A**, **B, D, E, F** Data shown were the mean ± SD and were from at least three independent experiments with each 3–4 replicates*.* **A-F** Data were analyzed by *T-*test, *p ≤ 0.05, **p ≤ 0.01, ***p ≤ 0.001, *ns.* not significant

We previously found that genetic deficiency of Viperin significantly decreased Mtb infection in the lungs and spleens of mice at 1 and 4 weeks, although no significant change of IFN-α, -β, or -γ production was found [18]. However, we observed that Viperin deficiency significantly increased IFN-γ, but not IFN-α or -β mRNA expression and production in BMDM at 24 and 48 h post Mtb infection (Fig. 1A). We also showed that Mtb infection at MOI of 0.5 and 1 could promote IFN-γ mRNA expression and production at 48 h (Fig. 1B). Viperin knockout was verified by the loss of Viperin mRNA and protein expressions at 48 h of Mtb infection in BMDM of Viperin deficient mice (Supplementary Fig. 1A, B). Next, we found that co-localization of macrophages marked by F4/80 and IFN-γ were increased in the lungs and spleens of Viperin deficient mice by immunofluorescence assay, indicating that the production of IFN-γ in macrophages from the lungs and spleen of Viperin deficient mice was significantly increased (Fig. 1C). In addition, Viperin deficiency significantly decreased Mtb infection at 48 and 72 h in BMDM (Fig. 1D). Neutralizing antibody of IFN-γ treatment could block IFN-γ production, but not IFN-γ mRNA expression, in Viperin deficient BMDM (Fig. 1E). Importantly, IFN-γ neutralizing restored the suppressed Mtb infection by Viperin deficiency (Fig. 1F). We did not observed cytotoxicity of IFN-γ neutralizing to the host cells detected by CKK8 kit (Supplementary Fig. 2A) and IFN-γ neutralizing did not directly influence the bacterial growth detected by CFU assay (Supplementary Fig. 2B). These results suggest that Viperin deficiency inhibits Mtb infection through promoting IFN-γ production in macrophages.

### Viperin negatively regulates IFN-γ production through TBK1 and IKKε signaling pathways to promote Mtb infection in macrophages

**Fig. 2:**
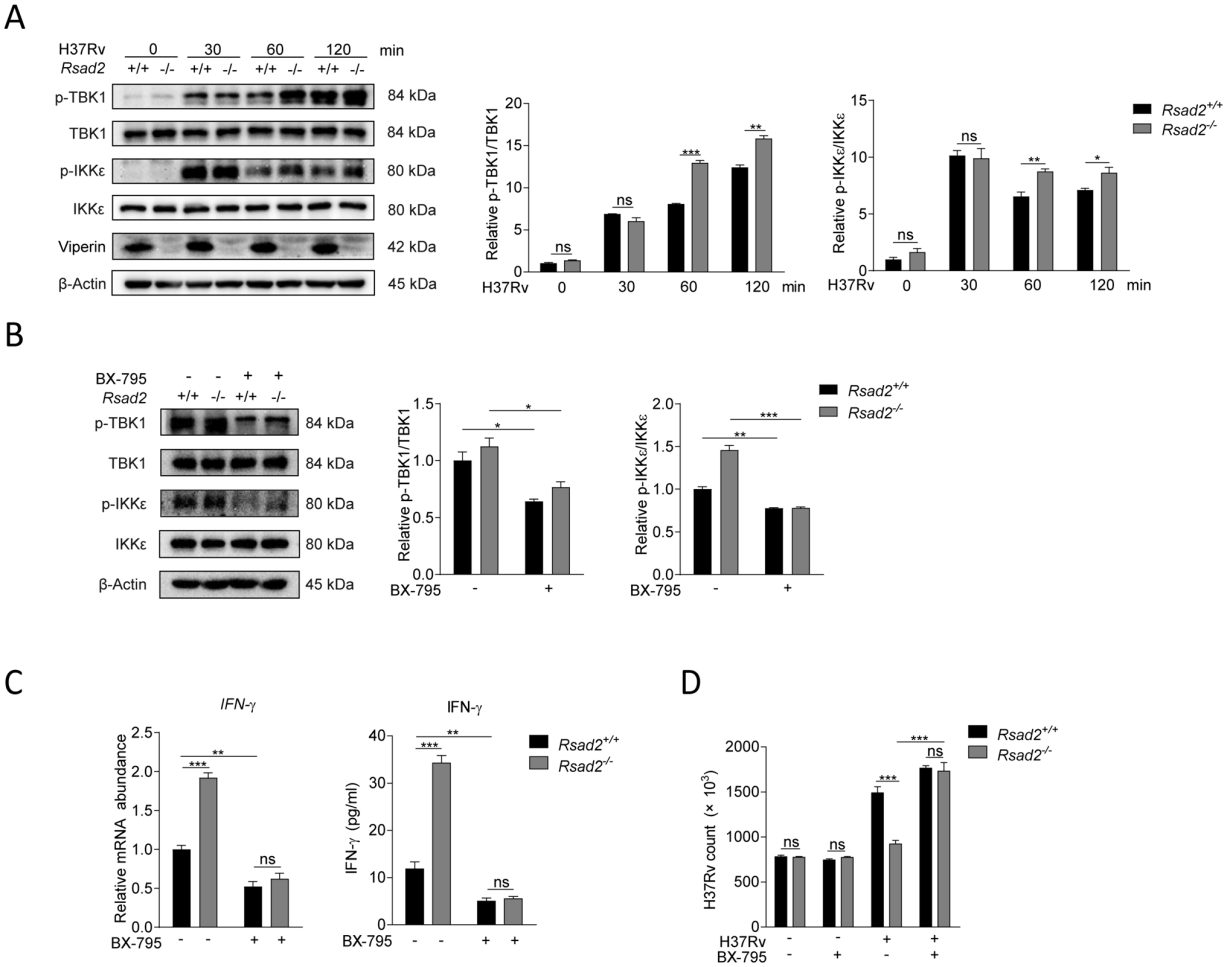
Viperin negatively regulates IFN-γ production through TBK1 and IKKε signaling pathways to promote Mtb infection in macrophages. **A** Phosphorylation levels of TBK1 and IKKε were detected by Western blot assay in *Rsad2*^+/+^ and *Rsad2*^−/−^ BMDMs infected with Mtb at MOI of 5 for the indicated times. **B** Phosphorylation levels of TBK1 and IKKε were detected by Western blot in *Rsad2*^+/+^ and *Rsad2*^−/−^ BMDMs that were pretreated with 2 μM BX-795 (TBK1/IKK-ε inhibitor) for 2 h and then infected with Mtb at MOI of 5 for 60 min. **C** Intracellular mRNA and secretion of IFN-γ were detected in *Rsad2*^+/+^ and *Rsad2*^−/−^ BMDMs pretreated with 2μM BX-795 for 2 h before 48 h of Mtb infection at MOI of 1 by qRT-PCR and ELISA analyses. **D** CFU counts were determined to analyze Mtb survival in *Rsad2*^+/+^ and *Rsad2*^−/−^ BMDMs pretreated with 2 μM BX-795 for 2 h at MOI of 5 for 48 h Mtb infection. **A**, **B** Data were subjected to densitometric analysis on basis of Western blot assay. β-Actin served as an internal reference. **C** Data were presented as fold change in mRNA abundance relative to that in untreated controls of *Rsad2*^+/+^ BMDMs. β-Actin served as an internal reference. **A**–**D** Western blot results were representative of three independent experiments with similar results. Data shown were the mean ± SD and are from at least three independent experiments with each 3–4 replicates*.* Data were analyzed by *T-*test, *p ≤ 0.05, **p ≤ 0.01, ***p ≤ 0.001, *ns.* not significant

TBK1-IKKε-IRF3 axis is an important signaling pathway to regulate IFN-γ production [8, 9]. We have detected key elements of the TBK1-IKKε-IRF3 signaling pathway and found that Viperin deficiency significantly promoted phosphorylation of TBK1 and IKKε at 60 and 120 min post Mtb infection in BMDM (Fig. 2A). The knockout effect of Viperin has been identified by the lack of Viperin expressions at 30, 60 and 120 min of Mtb infection in BMDM of Viperin deficient mice (Supplementary Fig. 1C). BX-795 is a potent TBK1 inhibitor, blocking activation of both TBK1 and IKKε. We treated Mtb infected-BMDM with BX-795 for 120 min, and the result showed that BX-795 treatment inhibited Viperin deficiency increased phosphorylation of TBK1 and IKKε (Fig. 2B). BX-795 treatment could also suppress Viperin deficiency promoted IFN-γ production in Mtb infected BMDM at 48 h (Fig. 2C). Consistently, Viperin deficiency reduced Mtb infection was restored by BX-795 treatment at 48 h post infection (Fig. 2D). BX-795 did not display cytotoxicity to the host cells detected by CKK8 kit (Supplementary Fig. 2C) and did not directly influence Mtb infection detected by CFU assay (Supplementary Fig. 2D). These results demonstrated that Viperin negatively regulates IFN-γ production through TBK1 and IKKε signaling pathways to promote Mtb infection in macrophages.

### Viperin suppresses Mtb-induced IFN-γ production via IRF3 phosphorylation in macrophages

**Fig. 3:**
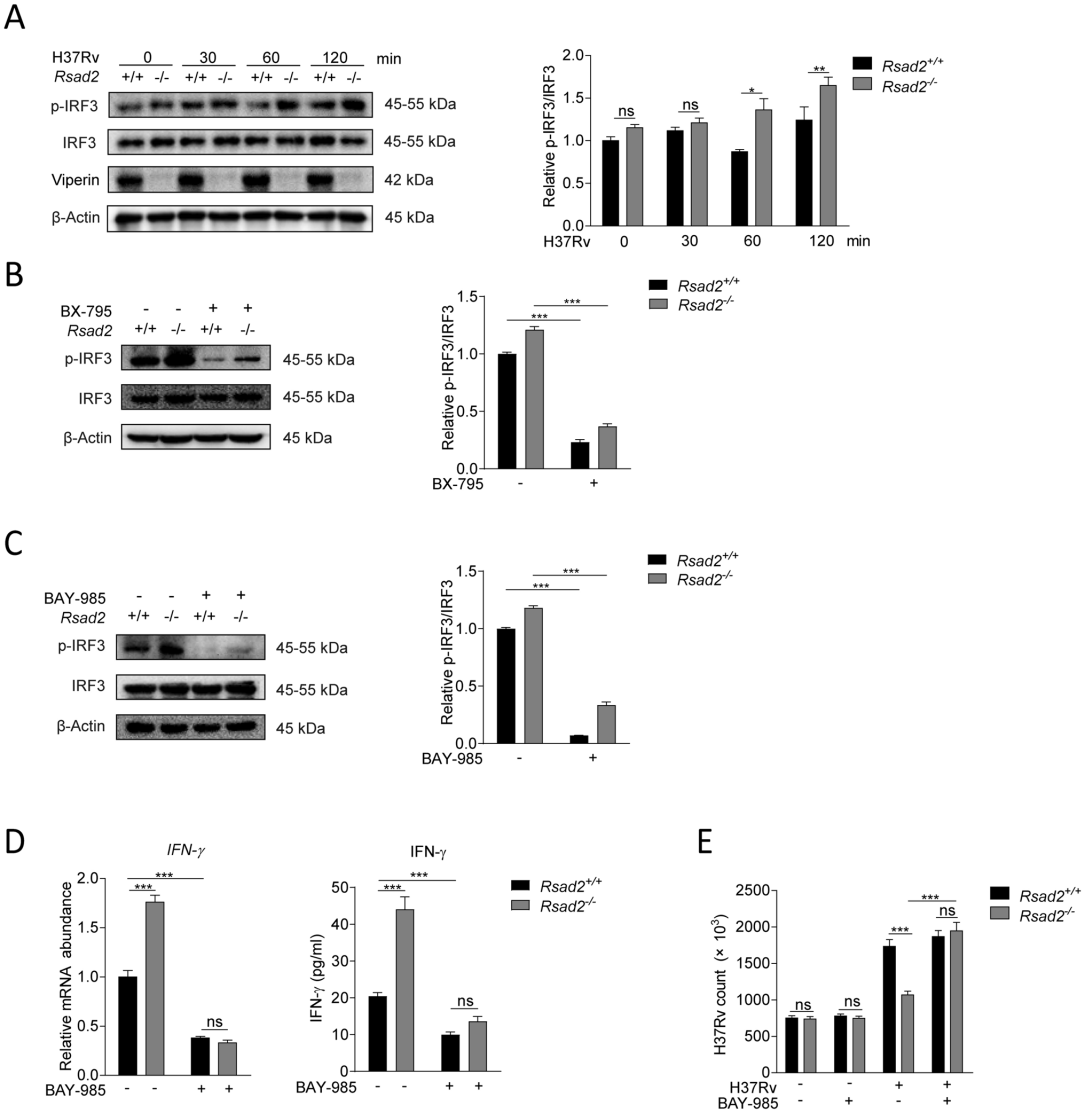
Viperin suppresses Mtb-induced IFN-γ production via IRF3 phosphorylation in macrophages. **A** Phosphorylation level of IRF3 was detected by Western blot assay in *Rsad2*^+/+^ and *Rsad2*^−/−^ BMDMs with Mtb infection at MOI of 5 for the indicated times. **B**,** C** Phosphorylation level of IRF3 was detected by Western blot assay in *Rsad2*^+/+^ and *Rsad2*^−/−^ BMDMs pretreated with B 2 μM BX-795 for 2 h and **C** with 200 nM BAY-985 (IRF3 inhibitor) for 18 h at Mtb infection at MOI of 5 for 60 min. **D** Intracellular mRNA and secretion of IFN-γ were detected in *Rsad2*^+/+^ and *Rsad2*^−/−^ BMDMs pretreated with 200 nM BAY-985 for 18 h upon Mtb infection at MOI of 1 for 48 h by qRT-PCR and ELISA analyses. **E** CFU counts were determined to analyze Mtb survival at MOI of 5 after 48 h of infection in *Rsad2*^+/+^ and *Rsad2*^−/−^ BMDMs pretreated with 200 nM BAY-985 for 18 h. **A**–**C** Data were subjected to densitometric analysis on basis of Western blot assay. β-Actin served as an internal reference. Data presented were from one of at least three independent experiments with similar results. **D** Data were presented as fold change in mRNA abundance relative to that in untreated controls of *Rsad2*^+/+^ BMDMs. β-Actin served as an internal reference. **A**–**E** Western blot results were representative of three independent experiments with similar results. Data shown were the mean ± SD and are from at least three independent experiments with each 3–4 replicates*.* Data were analyzed by *T-*test, *p ≤ 0.05, **p ≤ 0.01, ***p ≤ 0.001, *ns.* not significant

IRF3 is the downstream element of TBK1-IKKε signaling pathway and phosphorylation of IRF3 is the key factor to its activation and IFNs production [8, 9]. We have found that phosphorylation of IRF3 was significantly increased at 60 and 120 min of Mtb infection in Viperin deficient BMDM (Fig. 3A). As expected, BX-795 not only blocked TBK1 and IKK-ε, but also suppressed phosphorylation of IRF3 at 60 min of Mtb infection, which is detected by Western blot assay (Fig. 3B). Furthermore, we pretreat BMDM with BAY-985, an inhibitor of IRF3, and found that BAY-985 treatment inhibited phosphorylation of IRF3 in Mtb infected BMDM at 60 min (Fig. 3C). IFN-γ production increased by Viperin deficiency was completely blocked by Bay-985 treatment in Mtb infected BMDM at 48 h (Fig. 3D). Bay-985 treatment could restore Mtb infection in Viperin deficient BMDM at 48 h of infection (Fig. 3E). Also, Bay-985 did not display cytotoxicity to the BMDM detected by CKK8 kit (Supplementary Fig. 2C) and did not directly influence Mtb infection detected by CFU assay (Supplementary Fig. 2D). These results indicated that Viperin suppresses IFN-γ production through IRF3 phosphorylation downstream of TBK1-IKKε signaling pathways to promote Mtb infection in macrophages.

### Viperin inhibits IFN-γ production to facilitate Mtb infection by JAK1 phosphorylation

**Fig. 4:**
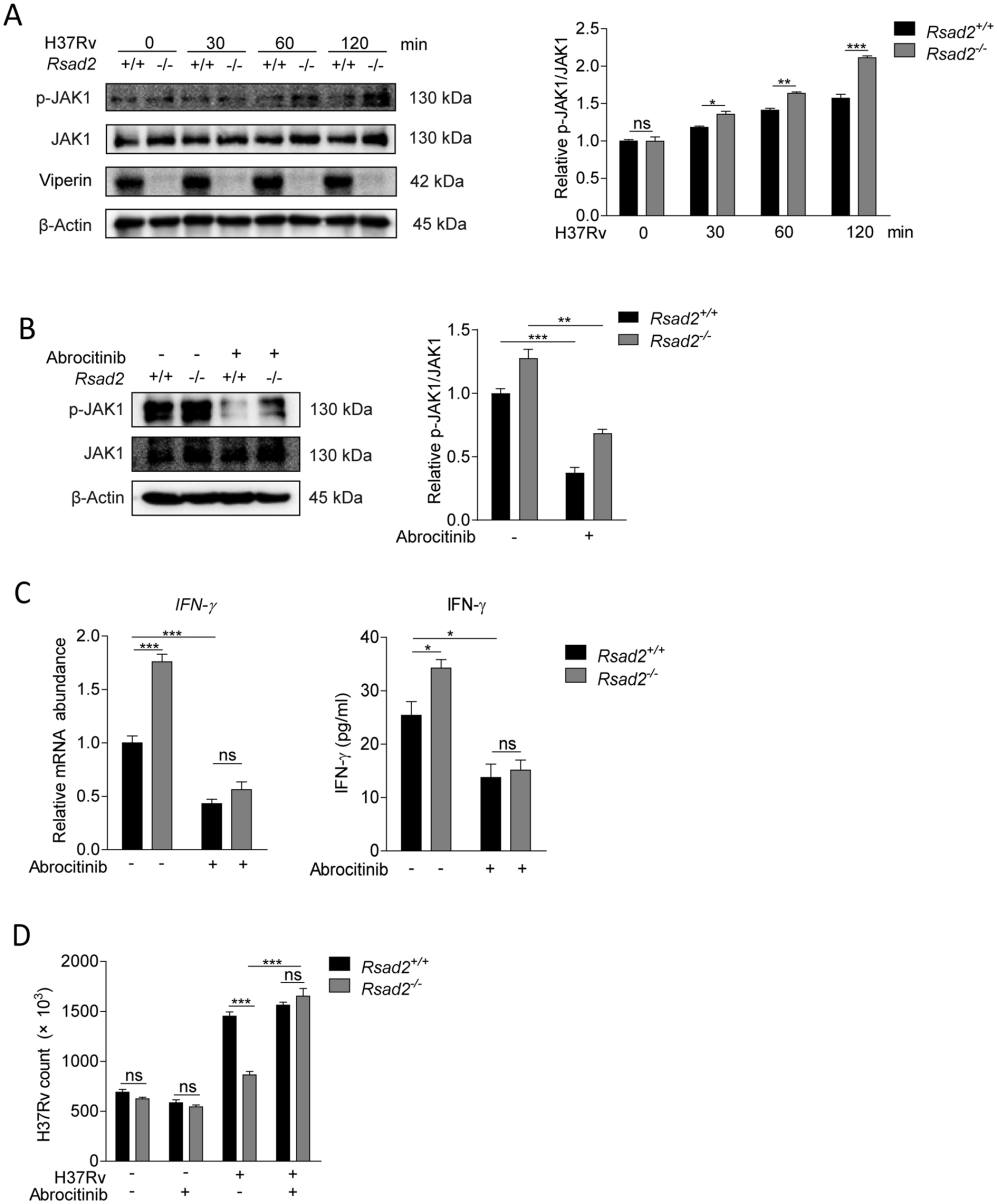
Viperin inhibits IFN-γ production to facilitate Mtb infection by JAK1 phosphorylation. **A** Phosphorylation level of JAK1 was detected by Western blot assay in *Rsad2*^+/+^ and *Rsad2*^−/−^ BMDMs with Mtb infection at MOI of 5 for the indicated times. **B** Phosphorylation level of JAK1 was detected by Western blot assay in *Rsad2*^+/+^ and *Rsad2*^−/−^ BMDMs that were pretreated with 200 nM Abrocitinib (JAK1 inhibitor) for 18 h and then infected with Mtb at MOI of 5 for 60 min. **C** Intracellular mRNA and secretion of IFN-γ were detected in *Rsad2*^+/+^ and *Rsad2*^−/−^ BMDMs pretreated with 200 nM Abrocitinib for 18 h before Mtb infection at MOI of 1 for 48 h by qRT-PCR and ELISA analyses. **D** CFU counts were determined to analyze Mtb survival at MOI of 5 for 48 h infection in *Rsad2*^+/+^ and *Rsad2*^−/−^ BMDMs that were pretreated with 200 nM Abrocitinib for 18 h. **A**, **B** Data were subjected to densitometric analysis on basis of Western blot assay. β-Actin served as an internal reference. **C** Data were presented as fold change in mRNA abundance relative to that in untreated controls of *Rsad2*^+/+^ BMDMs. β-Actin served as an internal reference. **A–D** Western blot results were representative of three independent experiments with similar results. Data shown were the mean ± SD and are from at least three independent experiments with each 3–4 replicates*.* Data were analyzed by *T-*test, *p ≤ 0.05, **p ≤ 0.01, ***p ≤ 0.001, *ns.* not significant

Binding of IFN-γ to its receptors can activate JAK-STAT signaling pathway to exert host defense function [10]. The JAK-STAT signaling pathway could also lead to the production of IFNs thus to form a loop [11]. We found that Viperin deficiency significantly promoted phosphorylation of JAK1 at 30, 60 and 120 min of Mtb infection in BMDM (Fig. 4A). However, protein expression of interferon regulatory factor 9 (IRF9), another critical molecular of JAK-STAT signaling, did not change in Viperin deficient BMDM (Supplementary Fig. 3). Treatment of Abrocitinib, a potent JAK1 inhibitor, could remarkably suppress phosphorylation of JAK1, at 60 min of Mtb infection (Fig. 4B). It could inhibit IFN-γ mRNA expression and production promoted by Viperin deficiency (Fig. 4C), and restored Mtb infection suppressed by Viperin deficiency (Fig. 4D). These effects of Abrocitinib did not rely on its cytotoxicity to the BMDM (Supplementary Fig. 2C) and the direct influence on Mtb infection (Supplementary Fig. 2D). These results suggest that Viperin suppresses JAK1 phosphorylation to inhibit IFN-γ production and facilitate Mtb infection in BMDM.

### Viperin deficiency promoted IFN-γ production to inhibit Mtb infection through STAT1 activation

**Fig. 5:**
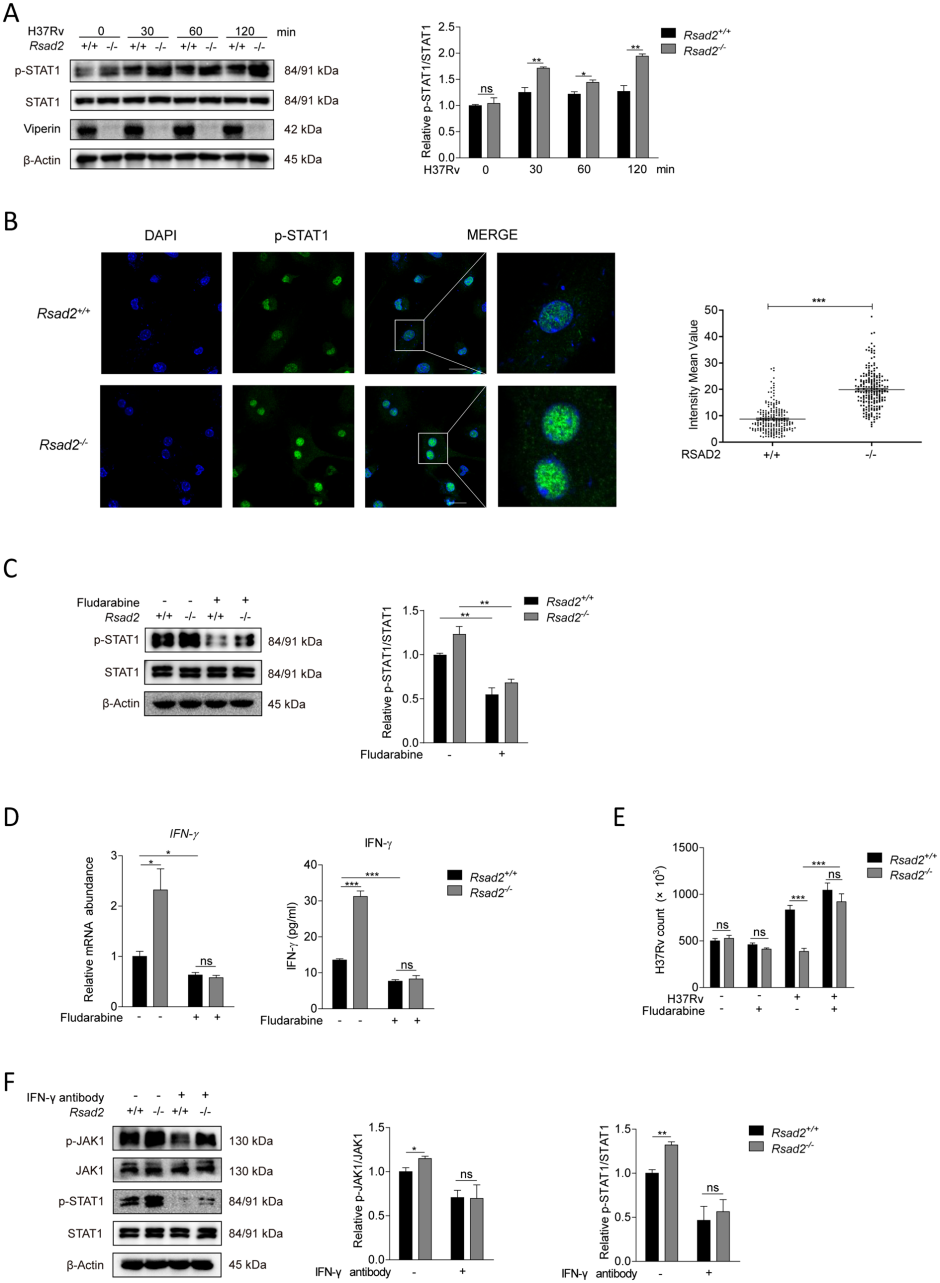
Viperin deficiency promoted IFN-γ production to inhibit Mtb infection through STAT1 activation. **A** Phosphorylation level of STAT1 was detected by Western blot assay in *Rsad2*^+/+^ and *Rsad2*^−/−^ BMDMs infected with Mtb at MOI of 5 for the indicated times. **B** *Rsad2*^+/+^ and *Rsad2*^−/−^ BMDMs infected with Mtb at MOI of 5 for 2 h. Phosphorylation level of STAT1 was detected with Alexa Fluor 488 (green) by immunofluorescence confocal microscope assay. DAPI (blue) was used to stain for the nuclear of the cells. Fluorescence intensity mean value of p-STAT1 Alexa Fluor 488 was detected in 200 cells in the nuclear of each group. Scale bar: 10 μm. **C** Phosphorylation level of STAT1 was detected by Western blot assay in *Rsad2*^+/+^ and *Rsad2*^−/−^ BMDMs that were pretreated with 30 μM Fludarabine (STAT1 inhibitor) for 2 h and then infected with Mtb at MOI of 5 for 60 min. **D** Intracellular mRNA and secretion of IFN-γ were detected in *Rsad2*^+/+^ and *Rsad2*^−/−^ BMDMs pretreated with 30 μM Fludarabine for 2 h before Mtb infection at MOI of 1 for 48 h by qRT-PCR and ELISA analyses. **E** CFU counts were determined to analyze Mtb survival at MOI of 5 after 48 h of infection in *Rsad2*^+/+^ and *Rsad2*^−/−^ BMDMs that were pretreated with 30μM Fludarabine for 2 h. **F** Phosphorylation levels of JAK1 and STAT1 were detected by Western blot assay in *Rsad2*^+/+^ and *Rsad2*^−/−^ BMDMs that were pretreated for 2 h with 7.5 μg/mL IFN-γ antibody and then infected with Mtb at MOI of 5 for 60 min. **A**, **C**, **F** Data were subjected to densitometric analysis on basis of Western blot assay. β-Actin served as an internal reference. **D** Data were presented as fold change in mRNA abundance relative to that in untreated controls of *Rsad2*^+/+^ BMDMs. β-Actin served as an internal reference. **A–F** Western blot results were representative of three independent experiments with similar results. Data shown are the mean ± SD and are from at least three independent experiments with each 3–4 replicates*.* Data were analyzed by *T-*test, *p ≤ 0.05, **p ≤ 0.01, ***p ≤ 0.001, *ns.* not significant

We further investigate whether Viperin regulates STAT1 activation, which is the downstream factor of JAK1. Firstly, we found that Viperin deficiency significantly increased phosphorylation of STAT1 at 30, 60 and 120 min of Mtb infection in BMDM (Fig. 5A). Next, we observed that Viperin deficiency could promote translocation of phosphorylated STAT1, but not total STAT1 protein, from cytoplasm to nucleus by immunofluorescence confocal microscope assay, indicating that Viperin suppressed STAT1 activation at 2 h of Mtb infection in BMDM (Fig. 5B and Supplementary Fig. 4). We excluded IRF1 as a possible molecular mechanism regulating Viperin deficiency induced-IFN-γ production as the result showed that Viperin deficiency did not influence translocation of IRF1 from cytoplasm to nucleus detected by immunofluorescence confocal microscope assay (Supplementary Fig. 5). Fludarabine is a specific inhibitor of STAT1 activation. To further investigate the role of STAT1 in IFN-γ production and Mtb infection, cells were pretreated with fludarabine for 2 h in Viperin deficient BMDM. We observed that fludarabine treatment suppressed phosphorylation of STAT1 (Fig. 5C) and inhibited IFN-γ production at 60 min of Mtb infection at 48 h of Mtb infection in BMDM (Fig. 5D). Fludarabine treatment reversed Mtb infection reduced by Viperin deficiency in BMDM (Fig. 5E). These effects of Fludarabine did not rely on its cytotoxicity to the BMDM (Supplementary Fig. 2C) and the direct influence on Mtb infection (Supplementary Fig. 2D). Furthermore, we observed that neutralizing antibody of IFN-γ treatment at 2 h of Mtb infection could inhibit phosphorylation of JAK1 and STAT1 caused by Viperin deficiency, suggesting that IFN-γ production and JAK-STAT signaling pathway can function on each other cyclically in BMDM (Fig. 5F). These results suggest that Viperin deficiency promotes IFN-γ production to inhibit Mtb infection through STAT1 activation.

### Viperin deficiency combined with INH treatment could further increase IFN-γ production and suppress Mtb infection

**Fig. 6:**
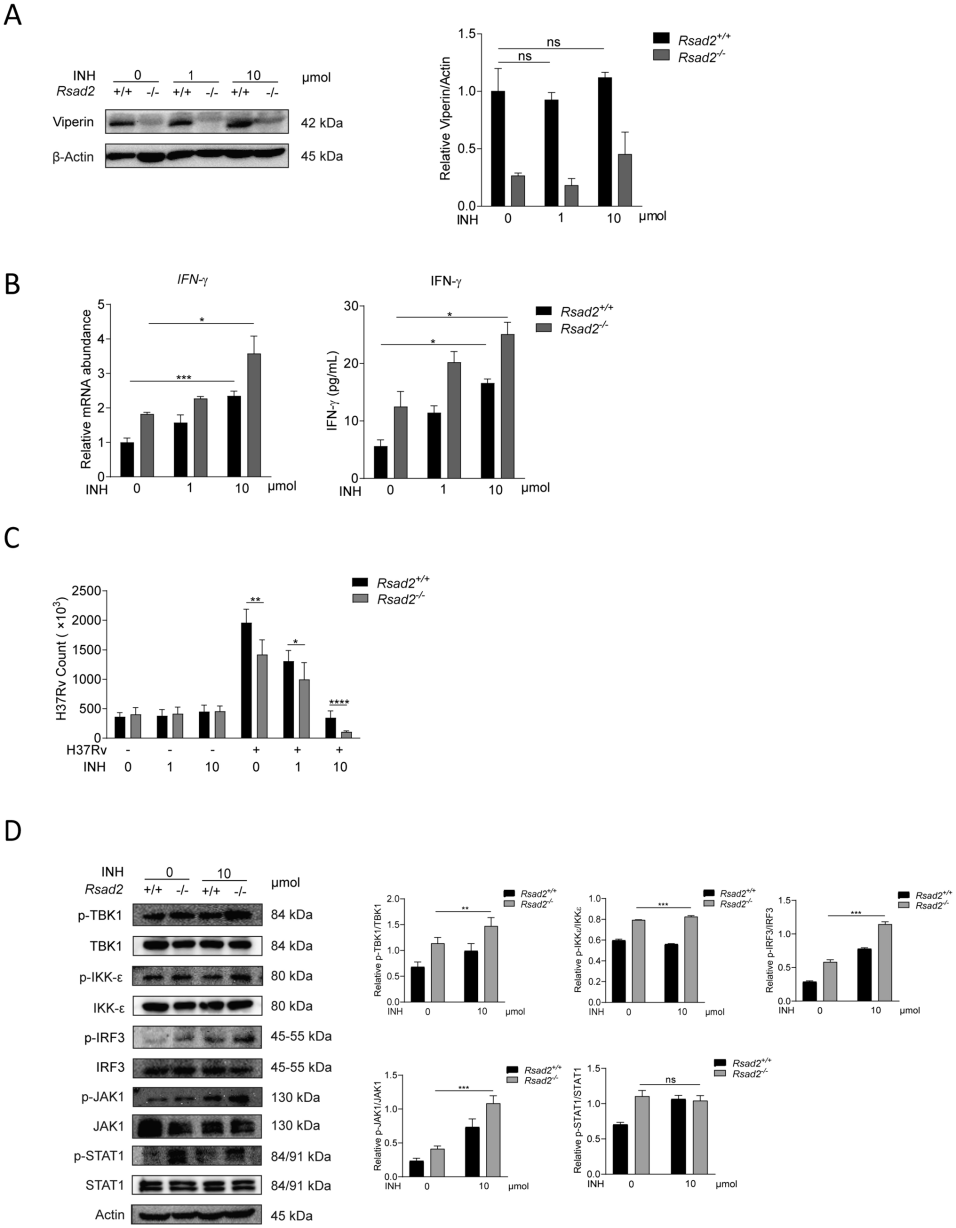
Viperin deficiency could further increase INH promoted IFN-γ production and suppress INH reduced Mtb infection. **A** Viperin expression was detected by Western blot assay in *Rsad2*^+/+^ and *Rsad2*^−/−^ BMDMs that were pretreated with 1 μM and 10 μM INH for 2 h and then infected with Mtb at MOI of 1 for 48 h. **B** Intracellular mRNA and secretion of IFN-γ were detected in *Rsad2*^+/+^ and *Rsad2*^−/−^ BMDMs pretreated with 1 μM and 10 μM INH for 2 h before Mtb infection at MOI of 1 for 48 h by qRT-PCR and ELISA analyses. **C** CFU counts were determined to analyze Mtb survival in *Rsad2*^+/+^ and *Rsad2*^−/−^ BMDMs that were pretreated with 1 μM and 10 μM INH for 2 h and then with Mtb infection at MOI of 5 for 48 h. **D** Total protein and phosphorylation levels of TBK1, IKK-ε, IRF3, JAK1 and STAT1 were detected in *Rsad2*^+/+^ and *Rsad2*^−/−^ BMDMs that were pretreated for 2 h with 10 μmoL INH and then infected for 60 min with Mtb at MOI of 5 by Western blot assay. **A** Data were subjected to densitometric analysis on basis of Western blot assay. β-Actin served as an internal reference. **B** Data were presented as fold change in mRNA abundance relative to that in untreated controls of *Rsad2*^+/+^ BMDMs. β-Actin served as an internal reference. **A–D** Western blot results were representative of three independent experiments with similar results. Data shown are the mean ± SD and are from at least three independent experiments with each 3–4 replicates*.* Data were analyzed by *T-*test, *p ≤ 0.05, **p ≤ 0.01, ***p ≤ 0.001, ns., not significant

Isoniazid (INH) is the first-line anti-TB drug, and INH treatment did not affect Viperin expression level (Fig. 6A). We treat Mtb-infected BMDM with a combination of a Viperin deficiency and the anti-TB drug isoniazid (INH), and found that IFN-γ production has been further increased compared with treatment with INH alone (Fig. 6B). Moreover, this combination also led to further reduction of Mtb infection in BMDM (Fig. 6C). Mechanically, we found that combination of a Viperin deficiency and INH could further increase phosphorylation of TBK1, IKKε, IRF3 and JAK1, but not STAT1 (Fig. 6D). In summary, these results showed that Viperin deficiency could further increase INH promoted IFN-γ production and suppress INH reduced Mtb infection through JAK-STAT signaling pathway.

## Discussion

Macrophages as the first-line in reacting to potent pathogens play versatile roles in innate immune response. During Mtb infection, macrophages respond to various cell products and secrete proinflammatory cytokines to activate the innate and acquired immune responses. Among these cell products, IFN-γ (originally called macrophage-activating factor) is one of the most important cytokines, playing a crucial role in antimicrobial effect by promoting antigen processing and presentation. Our previous studies have found that ISGs regulate the host immune response caused by Mtb infection [20, 21]. Among them, Viperin has been proven to negatively regulate the immune response towards Mtb infection. For example, Viperin led to the disruption of IRAK1-TRAF6-TAK1 complex and inhibition of TAK1-IKKα/β activation in macrophages, and Viperin impaired the activation of host defense function of dendritic cells (DCs) and DC-T cell cross talk during Mtb infection [18, 19]. It is worth noting that Viperin promoted Toll-like receptor 7 (TLR7)- and TLR9-mediated IRAK1 signaling and production of type I IFN by plasmacytoid dendritic cells (pDCs) to exert its antiviral function [17]. However, in this study, we found that Viperin inhibits type II IFN production in Mtb-infected macrophages. This opposite effect may be caused by different infectious agents that infect different types of cells. In addition, we previously found similar amounts of IFN-α, -β and –γ in the sera and lungs of Viperin deficient mice compared to those of control mice [18]. However, here we observed that in Mtb-infected macrophages, Viperin deficiency reduced IFN-γ production, but not IFN-α or -β. Consistently, Viperin deficiency promoted IFN-γ expression in macrophages of lungs and spleens of Mtb infected mice detected by immunohistochemically staining. Previously we found that Mtb infection was reduced in lungs and spleens of full-body Viperin knockout mice [17]. This result is not representative of the bactericidal mechanism produced by host macrophages in mice. Therefore, it is more reliable to use monocyte-specific knockout mice to verify whether Viperin could regulate immune response of macrophages in vivo. In this study, we demonstrated Viperin deficiency resulted in IFN-γ production at different time points and with different amounts of infection. The use of neutralizing antibody of IFN-γ can block the production of IFN-γ and restore Mtb infection caused by Viperin deficiency in macrophages. However, the specific mechanism of Viperin on IFN-γ production in Mtb-infected macrophages deserves further study.

The IFN-γ secretion by professional antigen presenting cells (APCs) is likely to be important in early host defense against Mtb infection, which is controlled by cellular signaling pathways and cytokines production. Upon Mtb infection in macrophages, multiple pattern recognition receptors (PRRs) and their downstream signaling pathways could be activated [22]. Our previous study demonstrated that Viperin could interrupt the interactions of IRAK1, TRAF6 and TAK1 and thus inhibit IRAK1-TRAF6-TAK1 complex, impairing activation of MAPK and NF-κB signaling, suppressing production of pro-inflammatory cytokines such as IL-1β, IL-6, TNF-α and NO production during Mtb infection in macrophages [18]. Here, we found that TBK1-IKKε-IRF3 axis activation is upregulated in Viperin deficient macrophages. The IRF3 signaling pathway is a central element of IFN-α and -β production. Studies have also shown that the generation of IFN-γ is closely related to IRF3 signaling pathway. For example, IRF3 contributes to IFN-γ/IFNGR signaling for expression of innate anti-viral proteins in macrophages [8]. Previous study has shown that Viperin recruited the adaptor molecules IRAK1 and TRAF6 to the lipid droplet to facilitate the ubiquitination of IRAK1 by TRAF6 that, in turn, promoted TLR7- and TLR9-mediated induction of the nuclear translocation of transcription factor IRF7, and subsequently increased the production of type I IFN by pDCs [17]. Consistent with the abovementioned study linking Viperin and IRF7 nuclear translocation, it was recently demonstrated that knockdown of Viperin reduced activation of NF-κB in carcinoma cells and downstream recruitment of IRF7 to the nucleus resulting in a decrease in IFN-β gene expression [23]. In addition, combination of mesenchymal stem cells (MSC) and tacrolimus (FK506) can inhibit TBK1-IRF3 phosphorylation, thus reducing IFN-γ production to extend graft survival [9]. Because IRF3 phosphorylation is associated with multiple upstream kinases, such as TBK1 and IKKε, we have used the inhibitors of TBK1-IKKε-IRF3 axis and found that they could inhibit Viperin deficiency promoted IFN-γ production, and restored Mtb infection in macrophages. These results demonstrated that Viperin can regulate host defense system by TBK1-IKKε-IRF3 axis mediated IFN-γ production against Mtb infection. Viperin did not affect the changes of IFN-α and -β production in our study, this may be the reason that there are still other signaling pathways that can regulate the changes of IFN-α and -β signaling pathways. According to other studies and our reports, Viperin can combine with important molecules in PRRs, such as IRAK1 and TRAF6, to regulate activation of downstream pathway, but whether Viperin could directly interact with upstream factor of TBK1-IKKε-IRF3 signaling pathway such as TRAF3 requires further investigation. In addition, interleukin (IL)-12 and IL-18 are the most notable cytokines that can promote IFN-γ synthesis in APCs, and the combination of IL-12 and IL-18 stimulation could further increase IFN-γ production[11]. On the side, IL-4, IL-10, transforming growth factor-βand glucocorticoids play negative roles in IFN-γ production [11]. However, whether these cytokines have effects on IFN-γ production in Mtb-infected macrophages remains unknown.

IFN-γ primarily functions through the JAK-STAT pathway, which is used by over 50 cytokines, growth factors, and hormones to affect immune response [24]. Specifically, IFN-γ could bind to its receptors and activate the JAK-STAT signaling pathways, to induce gene transcription within the target cells to exert host defense function [10]. In turn, the JAK-STAT signaling pathway could also lead to the production of IFN-γ [11]. It has been reported that activation of TBK1 and STAT1 played important roles in IFN-γ sensitivity in lung adenocarcinoma [25]. However, whether Viperin can regulate the downstream JAK-STAT signaling pathway through TBK1 and thus activate IFN-γ production needs to be further investigated. In our study, Viperin deficiency significantly increased levels of JAK1 and STAT1 phosphorylation, and neutralizing antibody of IFN-γ treatment could inhibit the phosphorylation. These results demonstrated that IFN-γ produced by the TBK1-IKKε-IRF3 signaling pathway can act on downstream JAK1 and STAT1 activation. The first wave of IFN-γ-induced transcription occurs quickly with IFN-γ production, and the transcription of many IFN-γ-responsive genes is controlled by a GAS element or an ISRE to further activate ISGs transcription [11]. Among these ISGs, IRF1 and MxA are most important ones have been shown with immune response regulatory function on Mtb infected macrophages in our previous studies [11, 21]. IRF1 could inhibit the mechanistic target of rapamycin (mTOR)/p70 S6 kinase (p70 S6K) cascade to suppress Mtb-infection in macrophages [20]. Another study revealed a novel role of MxA in down-regulating TAK1-IKKα/β-NF-κB signaling activation and production of antimicrobial inflammatory cytokines, facilitating Mtb infection in macrophages [21]. Another study found that other ISGs such as the host cytosolic RNA sensing molecules RIG-I-like receptor (RLR) signaling proteins RIG-I and MDA5, their common adaptor protein MAVS, and the RNA-dependent kinase PKR each independently inhibit Mtb growth in human cells [26]. IFITMs could mediate endosomal maturation and participate in the restriction of Mtb growth [27]. However, the role of many ISGs in immune regulation against TB infection remains unknown. In addition, functional STAT1 is crucial to host response to Mtb infection. For example, STAT1 knock out (KO) mice exerted defective of Tumor Necrosis Factor (TNF)-α, inducible NOS and IL-12 production, are highly susceptible to TB and succumb very rapidly to the disease. They developed multiple necrotic and granulomatous lesions in the lungs [28]. The association of STAT1 dimers with its associated co-activators such as cAMP response element binding protein (CREB) and p300 plays a vital role in IFN-γ-induced responses in Mtb-infected macrophages [29]. However, IFN-γ has been reported with function of pro- and anti-proliferative signals through STAT1-independent and STAT1-dependent pathways, respectively [11]. In our study, we have demonstrated that Viperin deficiency promoted IFN-γ relied on JAK1 and STAT1 activation, and this activation could further increase IFN-γ production, forming a cycle that continuously enhances pathway activation and ultimately helps macrophage to clear Mtb.

At present, many first-line drugs for the treatment of TB are considered to have side effects on patients, and it is reported that IFNs combined with first-line anti-TB drugs can be used in the treatment of MDR-TB patients [30]. As a first-line anti-TB drug, our previous studies have found that INH can exert its regulatory effect on Mtb infection through combining with other ISGs, including IRF1 and MxA. For example, INH treatment and IRF1 overexpression could further promote phosphorylation of mTOR and p70S6KA and reduce Mtb infection [20]. Treatment of INH with MxA silencing promoted TAK1-IKKα/β-NF-kB signaling pathway activation and inhibited Mtb infection [21]. In addition, IFN-γ has also been used as adjuvant therapy in TB patients when conventional therapy failed. Because Viperin knockout can produce IFN-γ in Mtb-infected macrophages, it is worth to investigate whether Viperin and INH can play a co-regulatory role in Mtb infection. Our study confirmed that INH does not affect the expression of Viperin protein in macrophages, but can affect the production of IFN-γ in a concentration-dependent manner. In addition, colony formation experiments further demonstrated that Mtb infection was significantly reduced by INH in the absence of Viperin in macrophages. Even so, since we have previously demonstrated that Viperin can play a pro-bacterial role through the production of other cytokines such as IL-6, TNF-α IL-1β, and NO [18], we cannot rule out antibacterial effect of these cytokines in the combined treatment of INH and Viperin deficiency.

**Fig. 7:**
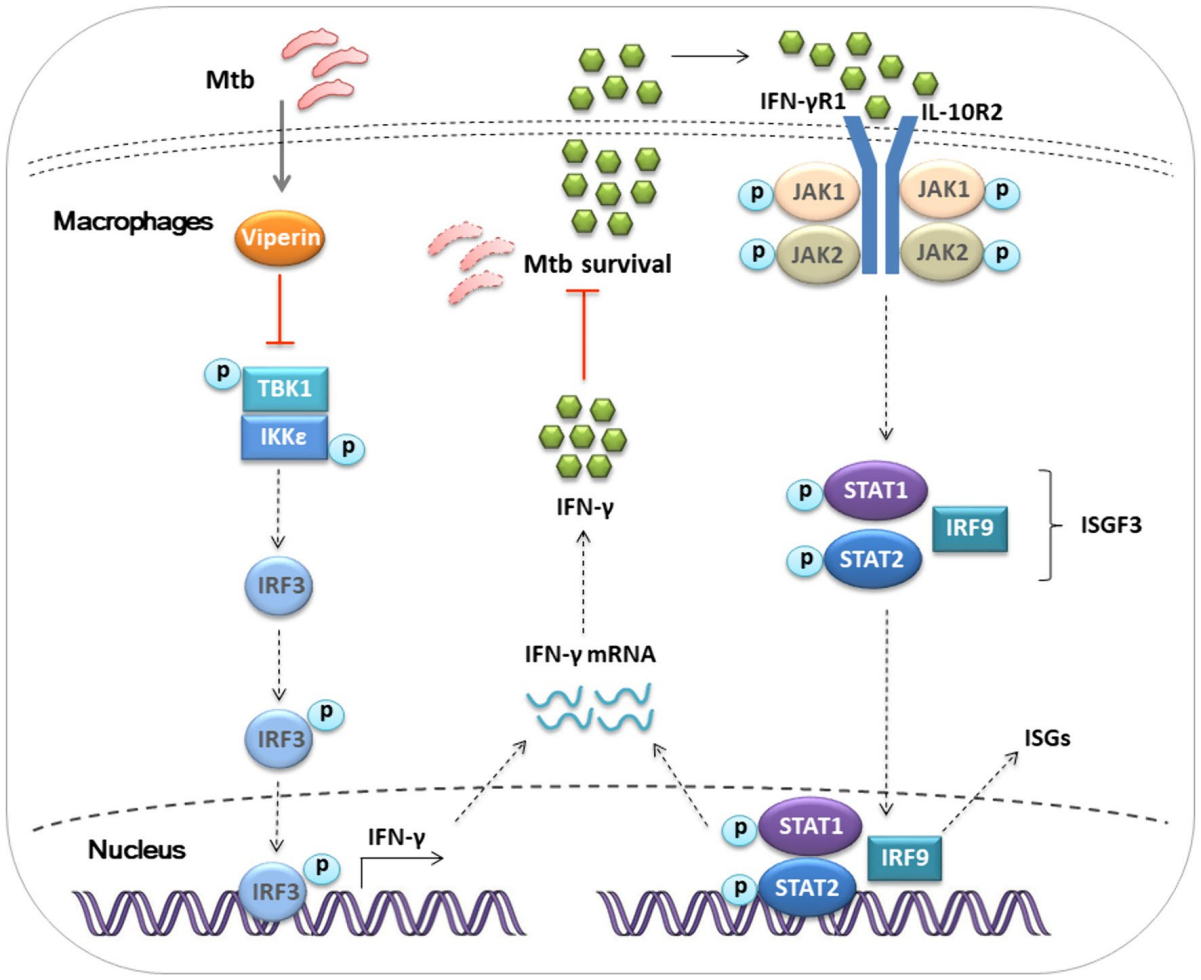
Viperin deficiency inhibits Mycobacterium tuberculosis infection by promoting TBK1-IKKε-IRF3-axis and JAK-STAT signaling induced IFN-γ production. See text for further details

In conclusion, it was preliminarily proved that Viperin deficiency results in promotion of IFN-γ production and inhibition of Mtb infection in macrophages and in lungs of mice in vivo. Mechanistically, Viperin deficiency could activate TBK1-IKKε-IRF3 signaling pathways, increase downstream IFN-γ production and suppress the intracellular survival of Mtb. Viperin deficiency can also upregulate JAK1-STAT1 signaling pathway, increase the entry of phosphorylation of STAT1 into the nucleus, and promote the production of downstream IFN-γ to achieve anti-TB effect (Fig. 7). Additionally, a combination of a Viperin deficiency and the anti-TB drug INH leads to further promotion of IFN-γ production and reduction of Mtb infection. Together with our previous research [18, 19], this study demonstrates that Viperin is a negative regulator of anti-tuberculosis immune response for host defense especially in APCs cells, including macrophages and DCs, proposes the potential of the combination of Viperin deficiency and first-line anti-TB drug, thus provides a novel host-directed therapy (HDT) for the treatment of TB.

### Supplementary Information

Below is the link to the electronic supplementary material.

Supplementary file1 (DOCX 3835 KB)

## Data Availability

The raw data supporting the conclusions of this article will be made available by the authors, without undue reservation.
