# Supplementary material for: Viperin inhibits interferon-γ production to promote Mycobacterium tuberculosis survival by disrupting TBK1-IKKε-IRF3-axis and JAK-STAT signaling
Source: Inflamm Res. 2024 Apr 16;73(6):897–913. doi: 10.1007/s00011-024-01873-w (PMC11106103; doi:10.1007/s00011-024-01873-w)
Supplement: Supplementary file 1 — Supplementary file1 (DOCX 3835 KB) [file 11_2024_1873_MOESM1_ESM.docx]

**Viperin inhibits interferon-γ production to promote *Mycobacteria tuberculosis* infection by disrupting TBK1-IKKε-IRF3-axis and JAK-STAT signaling**

Yao Liang^1,2^, Yun Liang^1,2^, Qi Wang^1,2^, Qianna Li^1,2^, Yingqi Huang^1,2^, Rong Li^1,2^, Xiaoxin Pan^1,2^, Linmiao Lie^1,2^, Hui Xu^1,2^, Zhenyu Han^1,2^, Honglin Liu^1,2^, Qian Wen^1,2^, Chaoying Zhou^1,2^, Li Ma^1,2^*, Xinying Zhou^1,2^*

^1,2^Institute of Molecular Immunology, School of Laboratory Medicine and Biotechnology, Southern Medical University, Guangzhou 510515, China.

^1,2^Key Laboratory of Infectious Diseases Research in South China (Southern Medical University), Ministry of Education.

**Short Title:** Viperin inhibits IFN-γ production to facilitate Mtb infection

*Corresponding author:

Dr. Xinying Zhou

Xinying Zhou, PhD, Institute of Molecular Immunology, School of Laboratory Medicine and Biotechnology, Southern Medical University, Guangzhou 510515, China. Phone: +86-20-61648553, Fax: +86-20- 61648555. E-mail: zxyforever@smu.edu.cn

Dr. Li Ma

Li Ma, PhD, Institute of Molecular Immunology, School of Laboratory Medicine and Biotechnology, Southern Medical University, Guangzhou 510515, China. Phone: +86-20-61648322, Fax: +86-20-61648322. E-mail: mali_61648322@smu.edu.cn

**SUPPLEMENTARY MATERIALS**

Figures S1-4

Tables S1-3

**
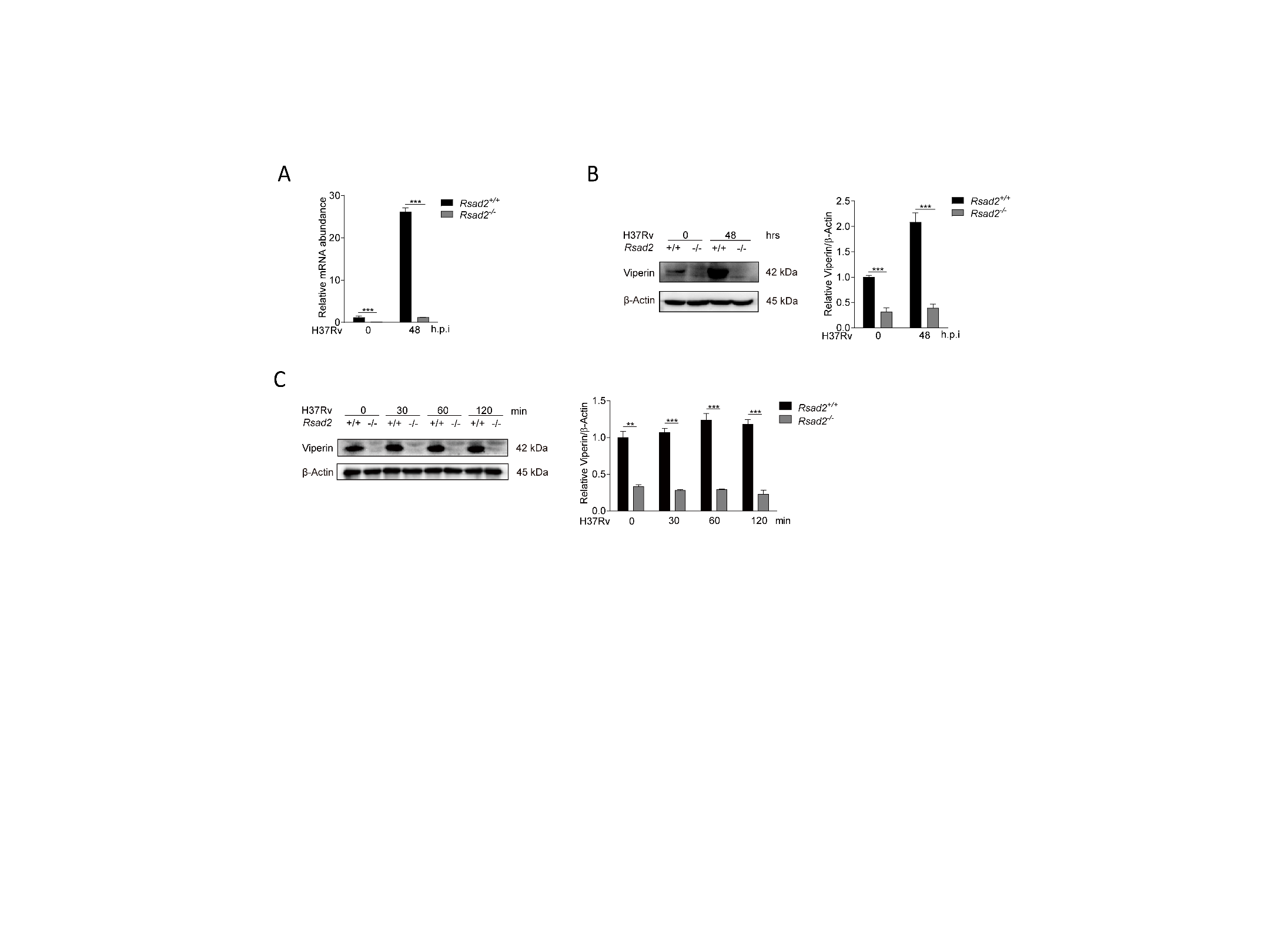
**

**Fig S1.** **Viperin expression in BMDM of *Rsad2*-deficient mice.**

**(A, B)** Intracellular mRNA (A) and protein levels(B) of Vipeirn were detected in *Rsad2*^+/+^ and *Rsad2*^-/-^ BMDMs after 48 hrs of Mtb infection at an MOI of 1 by qRT-PCR and Western blot analyses.

**(C)** Expression of Viperin at 0, 30, 60 and 120 minutes upon Mtb infection (MOI = 5) were determined by Western blot analysis.

**(A)** Data were presented as fold change in mRNA abundance relative to that in uninfected controls of *Rsad2*^+/+^ BMDMs. β-Actin served as an internal reference.

**(B, C)** Data were subjected to densitometric analysis on basis of Western blot. β-Actin served as an internal reference.

**(A-C)** Western blot results were representative of three independent experiments with similar results. Data shown were the mean ± SD and are from at least three independent experiments with each 3-4 replicates. Data were analyzed by T-test, **p≤0.01; ***p≤0.001.


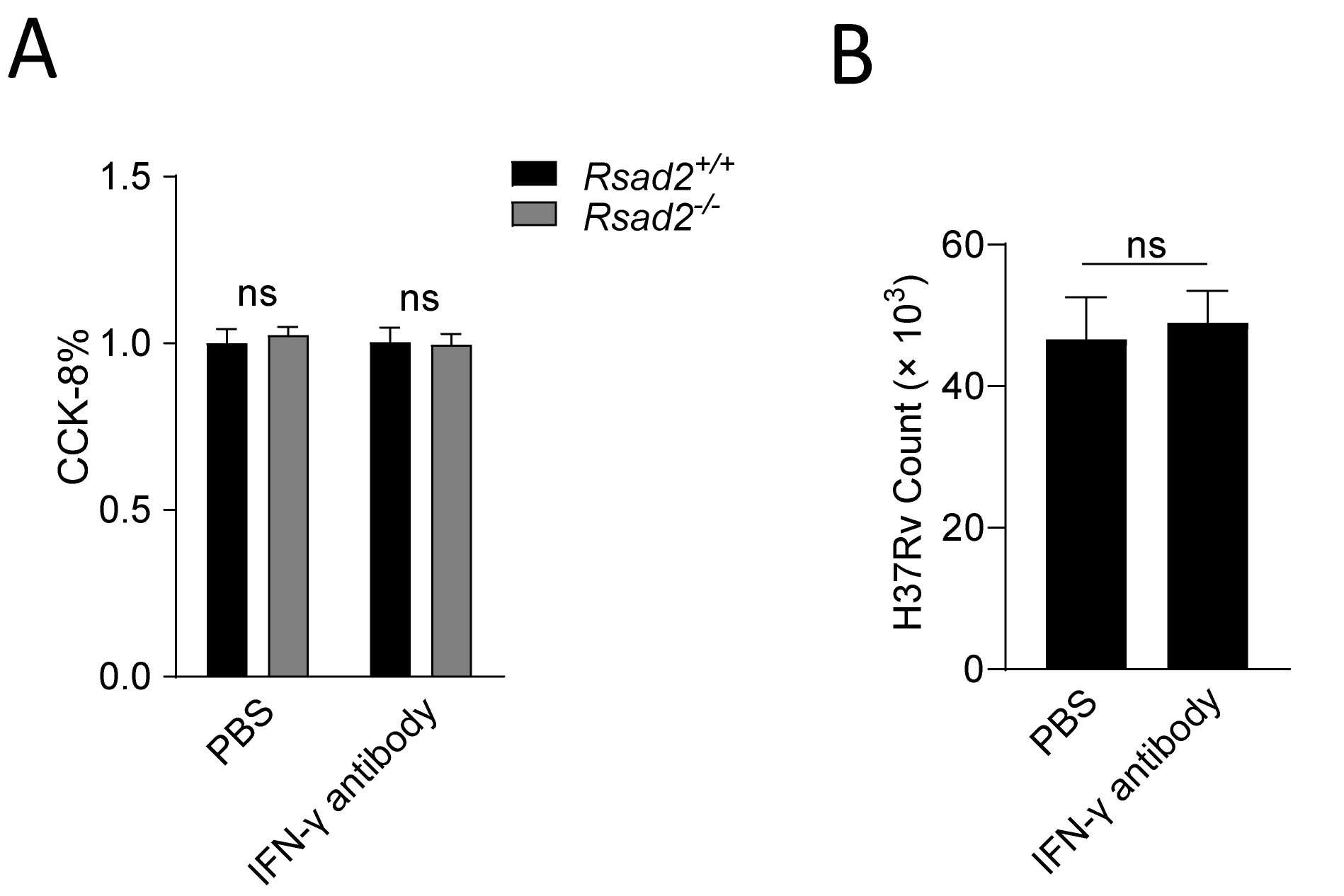

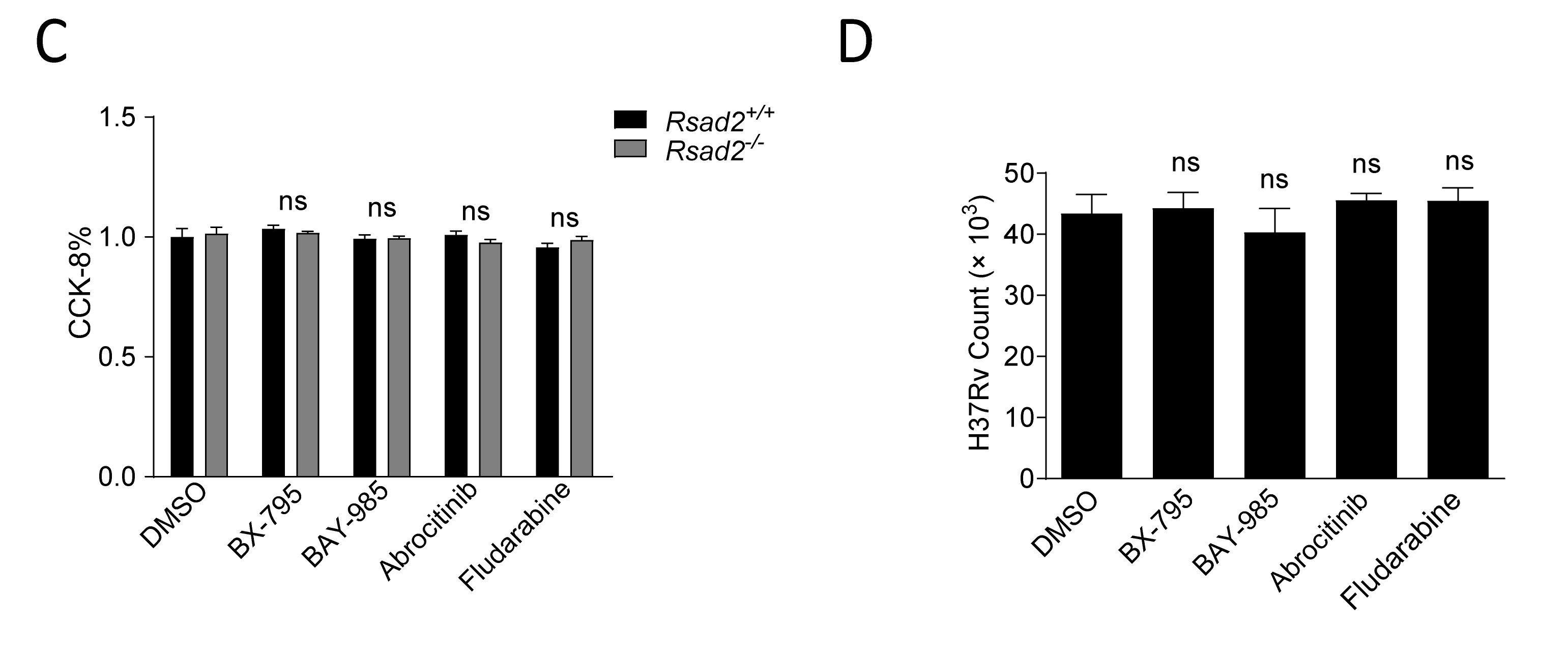


**Fig S2 Treatment of different reagents did not affect cytotoxicity of BMDM and Mtb growth.**

**(A)** *Rsad2*^+/+^ and *Rsad2*^-/-^ BMDMs were pretreated with IFN-γ antibody, cytotoxicity was detected by CCK-8 assay at 48 hrs. Treatment of PBS at the same concentration was normalized to 100%.

**(B)** The direct effect of IFN-γ antibody on Mtb infection was detected by CFU assay at 48 hrs.

**(C)** *Rsad2*^+/+^ and *Rsad2*^-/-^ BMDMs were pretreated for BX-795, BAY-985, Abrocitinib and Fludarabine, cytotoxicity was detected by CCK-8 assay at 48 hrs. Treatment of DMSO at the same concentration was normalized to 100%.

**(D)** The direct effect of inhibitors including BX-795, BAY-985, Abrocitinib and Fludarabine on Mtb infection was detected by CFU assay at 48 hrs.

**(A-D)** Data shown were the mean ± SD and are from at least three independent experiments with each 3-4 replicates. Data were analyzed by T-test, ns., not significant.

IFN-γ antibody: IFN-γ neutralizing antibody; BX-795: TBK1/IKKε inhibitor; BAY-985: IRF3 inhibitor; Abrocitinib: JAK1 inhibitor; Fludarabine: STAT1 inhibitor.

**
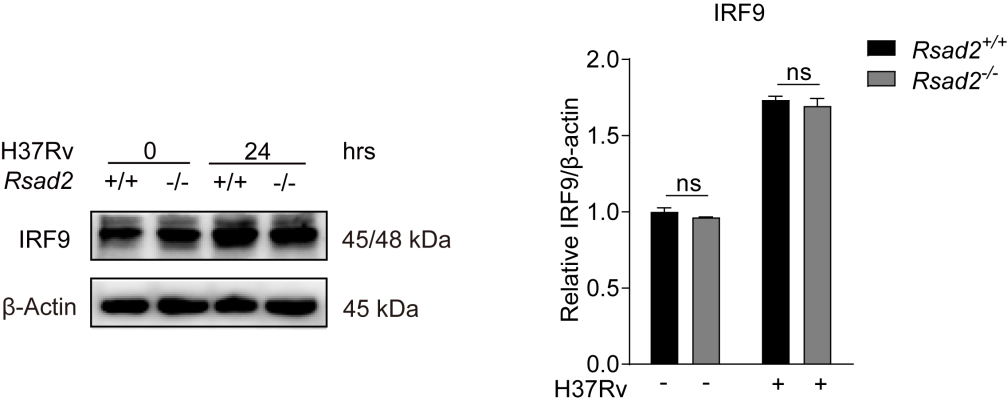
**

**Fig S3. Viperin deficiency did not affect protein expression of IRF9.**

Expression of IRF9 at 0, 24 hrs upon Mtb infection (MOI = 2) were determined by Western blot analysis. Data were subjected to densitometric analysis on basis of Western blot. β-Actin served as an internal reference.

Western blot results were representative of three independent experiments with similar results. Data shown were the mean ± SD and are from at least three independent experiments. Data were analyzed by T-test, ns., not significant.


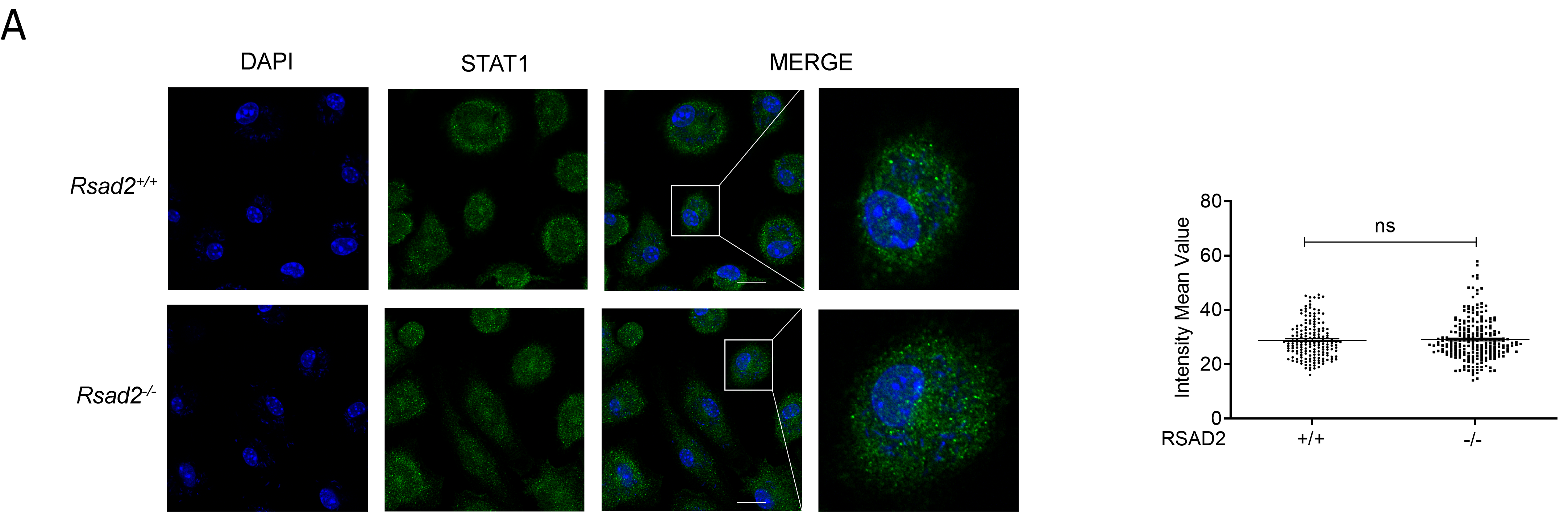


**Fig S4. Viperin deficiency did not affect translocation of total protein of STAT1 from cytoplasm to nucleus.**

**(A)** *Rsad2*^+/+^ and *Rsad2*^-/-^ BMDMs infected with Mtb (MOI=5) for 2 hrs. Levels of STAT1 was detected by Alexa Fluor 488 (green) with immunofluorescence confocal microscope assay. DAPI (blue) was used to stain for the nuclear of the cells. 200 cells were detected by fluorescence intensity mean value of STAT1 Alexa Fluor 488 in the nuclear in each group. Scale bar: 10 μm. Data were analyzed by *T-*test, ns., not significant.


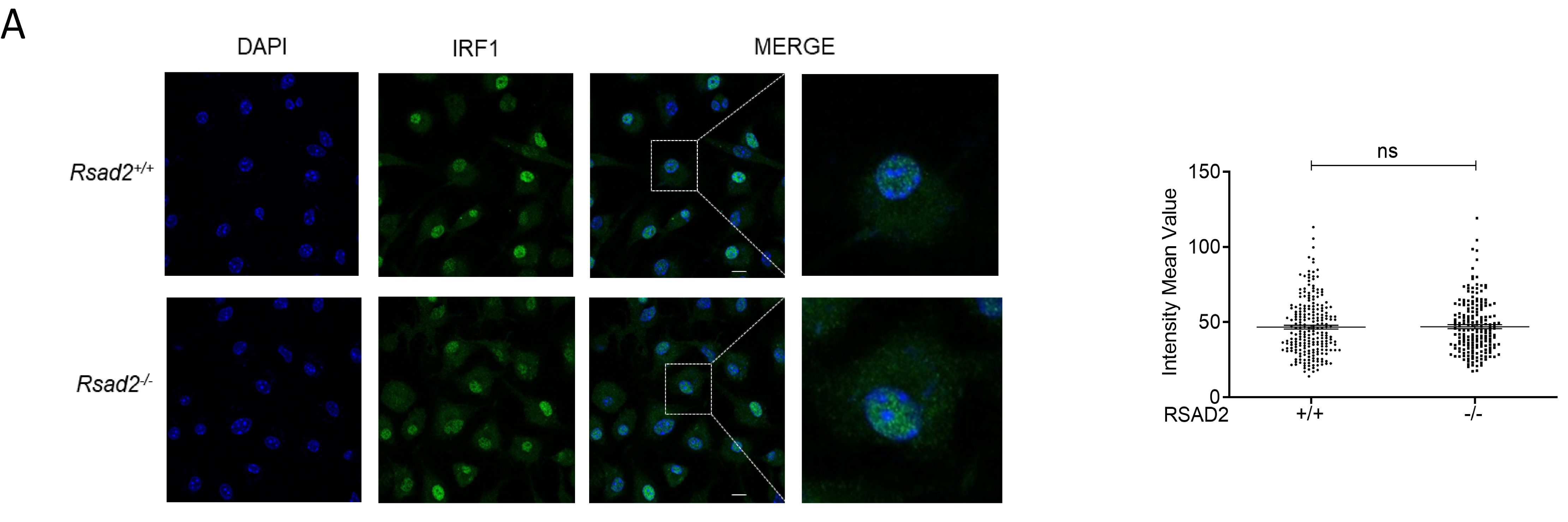


**Fig S5. Viperin deficiency did not affect translocation of total protein of IRF1 from cytoplasm to nucleus.**

**(A)** *Rsad2*^+/+^ and *Rsad2*^-/-^ BMDMs infected with Mtb (MOI=5) for 2 hrs. Levels of IRF1 was detected by Alexa Fluor 488 (green) with immunofluorescence confocal microscope assay. DAPI (blue) was used to stain for the nuclear of the cells. 200 cells were detected by fluorescence intensity mean value of IRF1 Alexa Fluor 488 in the nuclear in each group. Scale bar: 10 μm. Data were analyzed by *T-*test, ns., not significant.

**Table S1. List of reagents.**

| Reagent | **type** | **Approach** | **Source** |
| --- | --- | --- | --- |
| IFN-γ antibody | IFN-γ neutralizing antibody | 7.5μg/mL | Selleckchem, USA, A2105 |
| BX795 | TBK1/IKK-ε inhibitor | 2 μM, pretreatment 2 hrs | MCE, USA, HY-10514 |
| BAY-985 | IRF3 inhibitor | 200nM, pretreatment 18 hrs | MCE, USA, HY-133117 |
| Abrocitinib | JAK1 inhibitor | 200nM, pretreatment 18 hrs | MCE, USA,HY-107429 |
| Fludarabine | STAT1 inhibitor | 30μM, pretreatment 2 hrs | MCE, USA, HY-B0069 |
| INH | anti-TB drug isoniazid | 1, 10 μM, pretreatment 2 hrs | MCE, USA, HY-B0329 |

**Table S2. List of mouse genes and corresponding primers for qRT-PCR assay.**

| No. | Gene symbol | Full name | Primers (5’ - 3’) |
| --- | --- | --- | --- |
| 1 | Actin | Actin | F: CATTGCTGACAGGATGCAGAAGG  R: TGCTGGAAGGTGGACAGTGAGG |
| 2 | Rsad2 | Radical S-adenosyl methionine domain containing 2 | F: GGAAGGTTTTCCAGTGCCTCCT  R: ACAGGACACCTCTTTGTGACGC |
| 3 | [IFN-α](https://www.ncbi.nlm.nih.gov/gene/3440) | Interferon alpha | F: ATCCAGAAGGCTCAAGCCATCC  R: GGAGGGTTGTATTCCAAGCAGC |
| 4 | [IFN-β](https://www.ncbi.nlm.nih.gov/gene/3456) | Interferon beta | F: GCCTTTGCCATCCAAGAGATGC  R: ACACTGTCTGCTGGTGGAGTTC |
| 5 | [IFN-γ](https://www.ncbi.nlm.nih.gov/gene/3458) | Interferon gamma | F: CAGCAACAGCAAGGCGAAAAAGG  R: TTTCCGCTTCCTGAGGCTGGAT |

F: forward primer; R: reverse primer.

**Table S3. List of antibodies.**

| **Antibodies** | **Application** | **Dosage** | **Supplier** |
| --- | --- | --- | --- |
| β-actin | WB | 1:2000 | CST #8457 |
| Viperin [MaP.VIP] | WB | 1:50 | Abcam ab107359 |
| TBK1 | WB | 1:1000 | CST #3013 |
| Phospho-TBK1 (Ser172) | WB | 1:1000 | CST #5483 |
| IKK-ε | Neutralization | 1:8333 | CST #3416S |
| Phospho-IKK-ε (Ser172) | WB | 1:1000 | CST #8766S |
| IRF3 | WB | 1:1000 | Bioworld BS6921 |
| Phospho-IRF3 (Ser396) | WB | 1:1000 | Affintiy Biosciences AF2436 |
| JAK1 | WB | 1:1000 | CST #3344 |
| Phospho-JAK1 (Tyr1034/1035) | WB | 1:1000 | CST #74129S |
| STAT1 | WB  IF | 1:1000  1:400 | CST #14994 |
| Phospho-STAT1 (Tyr701) | WB  IF | 1:1000  1:300 | CST #9167S |
| Goat anti-Mouse IgG (H+L) Secondary Antibody HRP | WB | 1:2000 | Thermo 31430 |
| Goat anti-Rabbit IgG (H+L) Secondary Antibody HRP | WB | 1:2000 | Thermo 31460 |
| Donkey anti-Rabbit IgG (H+L) Highly Cross-Adsorbed Secondary Antibody, Alexa Fluor™ 488 | IF | 1:1000 | Thermo A21206 |
| F4/80 | IHC | 1:100 | Invitrogen MA5-16363 |
| IFN-γ | IHC | 1:200 | Abclonal A12450 |
| HRP-Goat Anti-Rabbit IgG antibody | IHC | 1:200 | 074-1506 |
| HRP-Goat Anti-Mouse IgG antibody | IHC | 1:200 | 074-1806 |

WB, Western Blot, IHC, Immunohistochemistry, IF, Immunofluorescence.
